# Patterns of cetacean vaginal folds yield insights into functionality

**DOI:** 10.1371/journal.pone.0175037

**Published:** 2017-03-31

**Authors:** Dara N. Orbach, Christopher D. Marshall, Sarah L. Mesnick, Bernd Würsig

**Affiliations:** 1 Department of Biology, Dalhousie University, Halifax, Nova Scotia, Canada; 2 Department of Marine Biology, Texas A&M University at Galveston, Galveston, Texas, United States of America; 3 Department of Wildlife and Fisheries Sciences, Texas A&M University, College Station, Texas, United States of America; 4 Southwest Fisheries Science Center, National Marine Fisheries Service, National Oceanic and Atmospheric Administration, La Jolla, California, United States of America; Universita degli Studi di Bari Aldo Moro, ITALY

## Abstract

Complex foldings of the vaginal wall are unique to some cetaceans and artiodactyls and are of unknown function(s). The patterns of vaginal length and cumulative vaginal fold length were assessed in relation to body length and to each other in a phylogenetic context to derive insights into functionality. The reproductive tracts of 59 female cetaceans (20 species, 6 families) were dissected. Phylogenetically-controlled reduced major axis regressions were used to establish a scaling trend for the female genitalia of cetaceans. An unparalleled level of vaginal diversity within a mammalian order was found. Vaginal folds varied in number and size across species, and vaginal fold length was positively allometric with body length. Vaginal length was not a significant predictor of vaginal fold length. Functional hypotheses regarding the role of vaginal folds and the potential selection pressures that could lead to evolution of these structures are discussed. Vaginal folds may present physical barriers, which obscure the pathway of seawater and/or sperm travelling through the vagina. This study contributes broad insights to the evolution of reproductive morphology and aquatic adaptations and lays the foundation for future functional morphology analyses.

## Introduction

The diversity and rapid evolution of male genitalia has been well-documented across many taxa, while the morphological variability of female genitalia has received comparatively little attention [1–2]. This lag in research efforts may reflect a male-biased view of the genital evolutionary process, easier accessibility of intromittent organs compared to vaginas, and erroneous assumptions regarding non-fluctuating vaginal forms [2–3]. As copulation is the most direct interaction between males and females [4], exploration of patterns of variation in vaginal morphology can provide insights into mechanisms of genital co-evolution. Sexual selection and sexual conflict are broadly accepted as the primary mechanisms driving male genital diversity [4–6]. In contrast, female genitalia have multiple functions in addition to copulation (e.g., sperm storage, oviposition, parturition) and evolve under constraints from both sexual and natural selection [4, 7]. While the genitalia of females are typically not as diverse as males, advances in analytical tools (e.g., scanning electron microscopy, transmission electron microscopy) have revealed extensive morphological variation in female genitalia within and across species [3, 8–9].

Female whales, dolphins, and porpoises (cetaceans) possess unusual folds of tissue in their vaginas, which are of unknown function(s) [10–11]. Vaginal folds are transverse protrusions of the vaginal wall into the vaginal lumen, with the distal tips often oriented caudally [12]. There is a broad diversity in vaginal fold morphology across cetaceans [13]. In many, but not all species, these vaginal folds are located in the cranial end of the vagina [14] and typically decrease in size cranially-to-caudally ([12; 15]; but see [16]). Similar vaginal structures are present in hippopotamuses (e.g., *Hippopotamus amphibius* [17]), which are the closest terrestrial relatives to cetaceans and also mate in the water. Vaginal folds appear to be unique to cetaceans and their closest relatives, the artiodactyls; no similar structures have been reported in non-cetacean marine mammals (phocids [18]; Australian sea lion, *Neophoca cinerea* [19]; California sea lion, *Zalophus californianus* [20]; Amazonian manatee, *Trichechus inunguis* [21]; sea otter, *Enhydra lutris* [22]).

Several alternative, but not mutually exclusive hypotheses have been proposed for the functions of cetacean vaginal folds, although none have been empirically tested. Vaginal folds have most often been suggested to function as adaptations for copulation in the marine environment [14; 23–27]. Seawater is lethal to common bottlenose dolphin sperm (*Tursiops truncatus* [28]), and presumably to the sperm of all cetaceans that mate in marine environments. Thus, vaginal folds, in addition to the cervix, may function to prevent seawater from contacting the ejaculate when the penis is inserted or withdrawn [14; 23–27]. Alternatively, vaginal folds might facilitate gestation and parturition. For example, vaginal folds have been hypothesized to counteract diving-related pressure changes and prevent the “expulsion of the fetus from the womb” [29], or to aid in parturition. Vaginal folds might aid in parturition by distending the reproductive tract [23; 30]. Orbach et al. [11] found that vaginal length and vaginal fold width were greater in pregnant compared to lactating common bottlenose dolphins (*T*. *truncatus*), although their sample size was too small for a robust comparison. They found negligible variation in vaginal morphology between sexually mature and immature post-mortem specimens.

Vaginal folds could also play a role in mating. For example, they may be adaptations to induce sperm competition [10]. Cetacean semen does not coagulate because males lack seminal vesicles and bulbourethral glands [31–32]. Semen coagulates are hypothesized to facilitate sperm retention and to block the pathway of sperm from rival males [33–34]. Accordingly, the vaginal folds of cetaceans might facilitate sperm retention and increase fertilization success in the absence of semen coagulation. Vaginal folds might constitute physical barriers to prevent the loss of semen [30; 32], or provide passageways for sperm transport along the fine longitudinal bands found on the vaginal folds [11], as observed in the cervices of some terrestrial mammals (goats and bovines [35–36]). Vaginal folds may also physically stimulate the penis during copulation and promote ejaculation, and possibly propel semen towards the uterine horns by muscle contractions to facilitate fertilization [30; 32].

Although the extensive variability in cetacean vaginal folds provide a rich and unique opportunity to explore the mechanisms of evolution of female genitalia, the underlying assumption of a morphological function has not been explored and is a necessary precursor. It is possible that vaginal folds are not adapted for specific functions and instead scale proportionally with body size and/or reflect phylogenetic history. The influence of body size on evolution and adaptations is well recognized across taxa [37–40]. Scaling relationships have been broadly applied to provide insights into underlying mechanisms of biological diversity [40]. Isometric patterns indicate that traits correlate with body size in contrast to allometric patterns, which indicate differential selection or investment in traits. While cetaceans demonstrate extensive morphological, physiological, and behavioral adaptations that enable them to thrive in the aquatic environment, body size is considered one of the most basic, but important adaptations [41–42]. The large body sizes of cetaceans have important implications for their swimming and diving efficiency, thermoregulation, life history attributes, sociality, and predation pressure [41–42]. This study sets the stage for future functional morphology analyses by first assessing the relationships of cetacean vaginal forms with body size while controlling for phylogenetic effects. Specifically, we test the hypotheses that vaginal length and cumulative vaginal fold length scale isometrically with body length, and that vaginal length predicts cumulative vaginal fold length in cetaceans.

## Materials and methods

### Data collection

Fifty-nine reproductive tracts (20 species and 6 families; S1 Table) were obtained opportunistically from cetaceans that stranded and died of natural causes. Entire reproductive tracts (external uro-genital slit to ovaries) were excised from fresh (< 24 hours post-mortem) or moderately decomposed deceased cetaceans and provided by marine mammal stranding networks throughout the coastal USA and from New Zealand. The reproductive tracts were frozen immediately and transferred to necropsy facilities located at Texas A&M University at Galveston or the National Oceanic and Atmospheric Administration’s (NOAA) Southwest Fisheries Science Center (La Jolla, California). Specimens were collected under National Marine Fisheries Service (NMFS) salvage permit letter and an institutional Convention on International Trade in Endangered Species of Wild Fauna and Flora permit (CITES). This study was exempt from an Institutional Animal Care and Use Committee (IACUC) authorization as the specimens were deceased upon acquisition and the salvage materials were authorized from appropriate government agencies. The marine mammal stranding networks provided data on the date and location of each stranding, sexual maturity state, and total body length. Total body lengths were straight-line measurements from the distal tip of the rostrum to the median notch on the trailing edge of the fluke [43]. Ideally, all specimens from any given species would represent only one age class. Due to the opportunistic nature of specimen acquisition and because no adult samples were obtained for six of the species included in this study, the specimens used were both sexually mature and immature females from a range of stranding locations (S1 Table). Minimal variation in vaginal morphology measurements has been found between sexual maturity states within at least one species (*T*. *truncatus* [11]).

**Fig 1:**
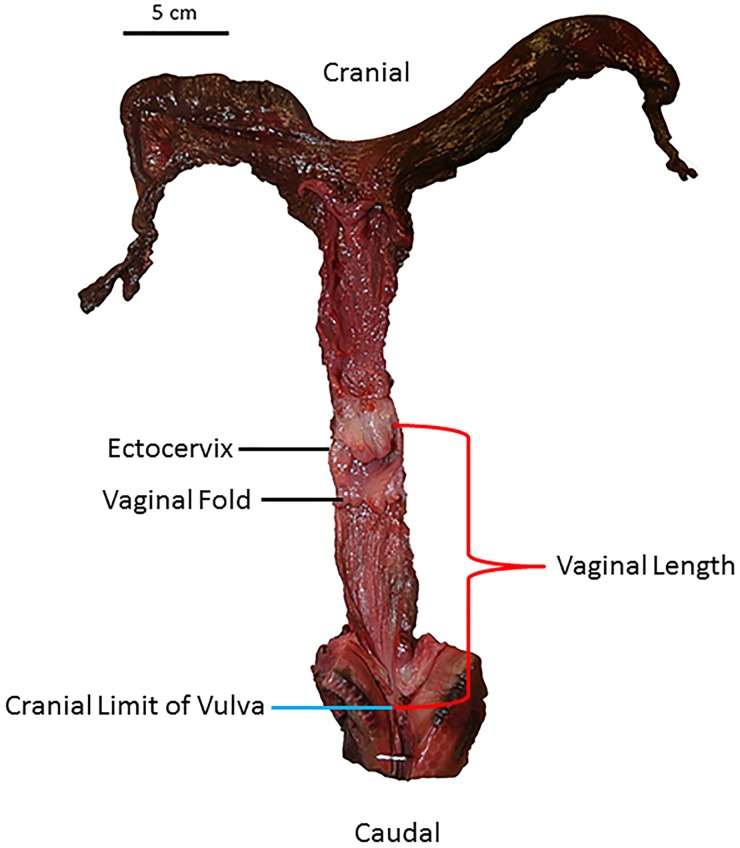
Vaginal length measurement of an adult female short-beaked common dolphin (*Delphinus delphis*). The reproductive tract is oriented in dorsal recumbency and splayed open. Vaginal length was measured with calipers along the midline of the dorsal vaginal wall. The measurement was taken in cranial to caudal orientation from the base of the ectocervix to the cranial limit of the vulva.

**Fig 2:**
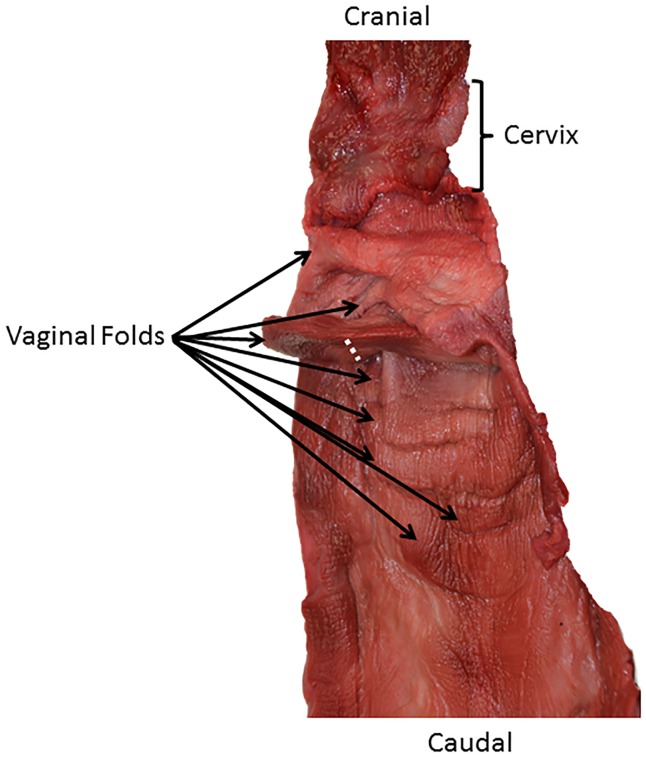
Vaginal fold length measurement of an adult female Pacific white-sided dolphin (*Lagenorhynchus obliquidens*). The black arrows point to eight vaginal folds in this specimen. Vaginal fold length was measured with a plastic scale positioned on the dorsal side of the fold. The cumulative measurements of vaginal fold length were straight lines from the base of the vaginal folds to their respective distal tips that projected into the lumen of the vagina. The hashed white line is positioned where one vaginal fold was measured.

The reproductive tracts were positioned in dorsal recumbency and bisected by a longitudinal midline incision from the clitoris to the external bifurcation of the uterine horns. Two measurements were collected following Orbach et al. [11]: 1) vaginal length and 2) cumulative vaginal fold length. The vaginal length was a cranial-to-caudal straight-line measurement from the base of the ectocervix (portion of the cervix in the vaginal lumen, where it met the vaginal wall) to the cranial limit of the vulva (Fig 1). The vaginal length was measured with calipers (+/- 0.02 mm) along the midline of the reproductive tract on the dorsal vaginal wall. The vaginal fold length was a straight-line measurement from the base of the vaginal fold (where it met the vaginal wall) to its distal tip that projected into the lumen of the vagina (Fig 2). The vaginal fold length was measured with a ruler positioned on the dorsal side of the vaginal fold. Cumulative vaginal fold lengths were summed for each specimen. A vaginal fold was defined as any folding of the vaginal wall at least 0.5 mm in length. Any vaginal fold less than 1 mm in length was rounded to 1 mm and included in the statistical analyses (but excluded from the count data in S1 Table).

### Analyses

Analyses were performed using the statistical program R and the ape and phytools packages [44–46]. Vaginal length, cumulative vaginal fold length, and total body length means were calculated for each species and base10 log-transformed to meet assumptions of a normal distribution and homogeneity of variance. The cetacean phylogenetic tree and branch lengths provided by McGowen et al. [47] were used to control for phylogenetic relatedness of species, and trimmed to remove species not in the database.

Scaling terminology follows Schmidt-Nielsen [37]. Since isometry refers to the slope of two variables (*x* and *y*), the predicted isometry depends on the dimensionality of these variables. That is, linear measurements of anatomy and body length scale isometrically with a slope of 1; each is a one dimensional measurement, whereas mass (a volume) scales isometrically to length to the third power. In this study, the predicted isometry for all length measurements to body length is a slope of 1. Reduced major axis regressions were used to account for error that could occur in measuring either *x* or *y* variable and to overcome scale dependence [48]. Models of the algebraic form log *y* = log *a* + *b* log *x* were fitted to the data. Three phylogenetic linear reduced major axis regressions were implemented using the RMA procedure in the R package phytools (phyl.RMA(x, y, tree, method = "lambda"); [46]). Mean vaginal lengths and mean cumulative vaginal fold lengths were regressed separately on mean total body lengths. For the third phylogenetically controlled regression of mean cumulative vaginal fold lengths on mean vaginal lengths, non-phylogenetically controlled residual values were first calculated of vaginal length on body length and of vaginal fold length on body length. To evaluate the fit of the linear phylogenetic RMA models to the data, R^2^ values were computed using the formula:

$$R^{2}=1-\frac{SS\;Residual}{SS\;Total},$$

where SS is the sum of squares. *T-*tests were used to examine significant deviations of the slope of the regression line (β_1_) from the isometric slope of 1 [49–50]. Isometry was inferred when there was no significant deviation between β_1_ and the isometric slope, negative allometry was inferred when there was significant deviation and β_1_ < 1, while positive allometry was inferred when there was significant deviation and β_1_ > 1 [49–50]. The 95% confidence intervals of β_1_ were also inspected to test for deviations from isometry. Thus a value of *P >* 0.05 fails to reject the null hypothesis of isometric scaling, corresponds with a predicted exponent within the 95% confidence intervals, and indicates a linear correlation between the log-transformed variables. A correlation structure (Pagel’s λ, derived from the Brownian motion model) that is robust to incomplete phylogenies was implemented to estimate the extent to which trait variation is related to phylogeny [51–52]. A λ value of 0 indicates phylogenetic independence, while a λ value of 1 indicates complete phylogenetic correlation [51–52]. We tested if the derived λ values were significantly better model fits than the null hypothesis (λ = 0) using log-likelihood ratio tests.

## Results

The 59 reproductive tracts examined varied widely in relative size, shape of folds, and number of folds. The number of vaginal folds ranged from one in common bottlenose dolphins (*T*. *truncatus*), long-beaked common dolphins (*Delphinus capensis*), and short-beaked common dolphins (*D*. *delphis*; Fig 1) to thirteen in harbor porpoises (*Phocoena phocoena*; N = 59, mean ± SD = 4 ± 2.8; S1 Table). The mean vaginal length of specimens was 21.1 cm (N = 59, SD = 15.3). The mean cumulative vaginal fold length was 51.5 mm (N = 59, SD = 47.5). All vaginal folds greater than 1 mm projected caudally towards the external vaginal opening. Directionality of projection could not be determined in vaginal folds less than 1 mm in length. In almost all specimens, vaginal folds were only present in the cranial half of the vagina. With the exception of some specimens in the genera *Phocoena*, *Kogia*, *Orcinus*, and *Lagenorhynchus*, vaginal folds generally decreased in length from the cranial to caudal direction.

**Fig 3:**
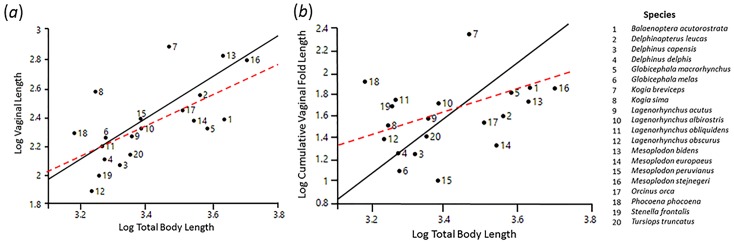
Regression of (A) vaginal length on total body length and (B) cumulative vaginal fold length on total body length. The solid black lines indicate the lines of best-fit from a phylogenetic reduced major axis regression, while the hashed red lines indicate the isometric slopes. N = 20 species.

**Fig 4:**
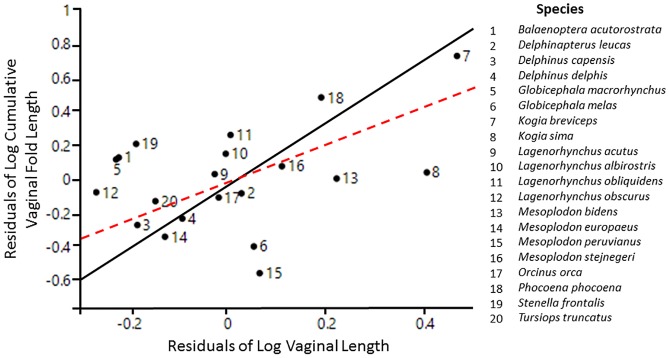
Regression of cumulative vaginal fold length on vaginal length. The non-phylogenetically controlled residuals of vaginal length on total body length and the non-phylogenetically controlled residuals of cumulative vaginal fold length on total body length were used. The solid black line indicates the line of best-fit from a phylogenetic reduced major axis regression, while the hashed red line indicates the isometric slope. N = 20 species.

Vaginal length scaled isometrically to body length (R^2^ = 0.426, *t* = 1.873, *df* = 17, *P* = 0.079; Table 1; Fig 3A) and a phylogenetic signal was present in the data (λ = 0.684; *P* = 0.002). In contrast, cumulative vaginal fold length showed significant positive allometry (R^2^ = 0.160, *t* = 3.596, *df* = 19, *P* = 0.002; Table 1; Fig 3B) in addition to less and non-significant phylogenetic signal (λ = 0.359; *P* = 0.22). Vaginal length was not a strong predictor of cumulative vaginal fold length (R^2^ = 0.093, *t* = 2.630, *df* = 19, *P* = 0.016, λ = 0.523; Table 1; Fig 4), although the relationship between the two variables showed some significant phylogenetic signal (λ = 0.523; *P* = 0.04).

**Table 1:** Scaling relationships among body length, vaginal length, and cumulative vaginal fold length.

Variable (Log vs Log)	*R*^2^	β_0_	β_1_	Lower 95% CI	Upper 95% CI	Predicted exponent	ƛ	P	P_Δlog(L)ƛ_
VL *vs*. BL	0.43	-2.35	1.40	0.95	1.85	1	0.68	0.08	0.002
VctFL *vs*. BL	0.16	-5.85	2.17	1.49	2.86	1	0.36	0.002	0.22
VFL_residuals_ *vs*. VL_residuals_	0.09	-0.05	1.80	1.16	2.44	1	0.52	0.02	0.04

VL = vaginal length; BL = body length; VFL = vaginal fold length; β_0_ = intercept; β_1_ = slope; CI = confidence interval; ƛ = phylogenetic signal; Δlog(L)ƛ = difference in log-likelihood ratios between observed ƛ and ƛ_0_. R^2^ calculated from linear phylogenetic reduced major axis regressions.

## Discussion

The scaling of reproductive tracts (how genitals change in size relative to changes in body size) has been studied for males, both within and among several taxonomic groups [49]. The genitalia of male mammals tend to show significant positive allometry (e.g., testes mass in Cape ground squirrels, *Xerus inauris* [50]; penis length in bats [53]; baculum size in harp seals, *Pagophilus groenlandicus* [54]; penis length in in Hottentot golden moles, *Amblysomus hottentotus* [55]; baculum size in muskrat, *Ondatra zibethicus* [56]), although scaling relationships may vary with the strength of pre- and post-copulatory sexual selection and/or mating systems [57–60]. However, few studies have investigated the scaling of female mammalian reproductive tracts. An isometric relationship was found with vaginal length in Hottentot golden moles [55], while no significant scaling patterns were found between body length and vaginal length or vaginal mass in Cape ground squirrels [50] or vaginal length in chimpanzees (*Pan troglodytes* [61]). Significant positive allometry was reported for vaginal length in Cape dune mole-rats (*Bathyergus suillus*); however, this pattern was exclusive to the breeding season [49]. Our finding, that vaginal length scales isometrically (Fig 3A), is congruent with other studies of female mammals and establishes a pattern of scaling of female reproductive organs of terrestrial and aquatic mammals by assessing trends across species. We advocate the need to broadly assess scaling patterns within phylogenies, as we found that variation in vaginal length across cetaceans is strongly connected to taxonomic relatedness (especially among species in the genus *Delphinus*). Since vaginal protrusions are uncommon in most mammals (with the exception of some artiodactyls), comparable studies of vaginal morphology scaling patterns in relation to vaginal lengths or body lengths are scant. Future studies that quantify variation in the vaginal morphology of artiodactyls and compare patterns with cetaceans will be particularly valuable in elucidating the function(s) of vaginal folds.

The extensive morphological variation observed in vaginal structures across cetaceans has not been documented in other mammalian orders. Cetacean vaginal folds not only varied in size, but also in number, positioning, and shape across species. Linear scaling with body size can be used as an explanatory factor for the observed diversity in vaginal length (Fig 3A). The significantly positive allometry of cumulative vaginal fold length to body length (Fig 3B), in addition to the low variance explained by the regression (16%) and non-significant phylogenetic signal, suggest there may be additional explanatory factors driving the diversity of vaginal fold lengths besides scaling patterns and phylogeny. Species with longer vaginal lengths tended to have disproportionately longer cumulative vaginal fold lengths (Fig 4). Since vaginal length and cumulative vaginal fold length varied independently of each other, and only vaginal length showed some significant phylogenetic signal, it appears that selective forces act differently on vaginal length and cumulative vaginal fold length in cetaceans. Accordingly, we suggest that the morphological diversity of vaginal folds reflect adaptations for specific functions. Two non-mutually exclusive functional hypotheses are discussed but not explicitly tested since this is outside the scope of this study and requires more experimental data: 1) the prevention of the incursion of seawater into the reproductive tract [14; 23–27] and 2) post-copulatory sexual selection [10–11].

The evolution of vaginal folds may reflect adaptive mechanisms developed to overcome challenges associated with living and mating in the marine environment. Vaginal length and cumulative vaginal fold length could reflect different mechanisms for the same function—to prevent the flow of seawater. Recognition that multiple morphological characteristics can serve one function (many-to-one mapping of form to function) is common among a wide range of vertebrates [62]. Since seawater is lethal to cetacean sperm [28], long vaginas may hinder the flow of water into the vaginal cavity simply due to the physical distances. Specifically, long vaginas may thereby hamper the incursion of water into the cranial part of the vagina (where semen is deposited) and the upper reproductive tract (where fertilization occurs and the fetus develops in the uterine horn). Vaginal folds can form deep crypts, which may function as physical barriers to seawater flow and might be particularly important in species with short vaginas.

However, there are several lines of evidence that do not support the functional hypothesis of preventing the flow of seawater into the vagina and instead support an alternative hypothesis related to post-copulatory sexual selection. For example, species with short vaginas often possessed short vaginal fold lengths (Fig 4). If vaginal folds function as physical barriers, then an inverse relationship between these two variables would be expected. Similarly, species with few vaginal folds had significantly shorter relative vaginal fold lengths compared to species with many vaginal folds (S1 Fig). Vaginal folds were usually concentrated at the cranial end of the vagina rather than distributed evenly through its length, although seawater could also be present in the caudal vagina. To prevent seawater from entering the vagina, it would be expected that vaginal folds would be greater in length at the caudal vagina (point of contact with seawater). Instead, vaginal folds generally decreased in length from the cranial to caudal direction. As it is commonly thought that all water (including freshwater) is lethal to mammalian sperm [63–64], the presence of vaginal folds in a river dolphin, which inhabits only freshwater environments, does not preclude the barrier to water functional hypothesis (baiji, *Lipotes vexillifer* [26]). However, if vaginal folds serve as physical barriers to water flow, it is unclear why other, non-cetacean marine mammals, which mate only in the water, also lack vaginal folds (e.g., phocids [18], Amazonian manatees [21], sea otters [22]). Collectively, these observations suggest that other factors are needed to explain the variability in the presence and development of vaginal folds in cetaceans.

Cetaceans may have other barriers which occlude seawater from entering their reproductive tracts and affecting osmoregulation. The lack of vaginal fold-like structures in non-cetacean marine mammals may indicate that the tight vaginal seal of the *labia minora* may be sufficient to prevent seawater and marine debris from entering the reproductive tract, including during non-mating contexts. Any seawater that overcomes the physical barrier of the *labia minora* may encounter additional challenges, which prevent entry into the cranial vagina or upper reproductive tract. For example, the hymeneal folds of pinnipeds and manatees are located more caudally in the vagina compared to cetacean vaginal folds and could potentially form a physical barrier [65]. The cervix functions as a critical barrier to the upper reproductive tract. The anti-microbial defenses and immune responses of the vagina and cervix, in addition to cervical mucus, can prevent movement of foreign bodies into the upper reproductive tract [66]. The thick cervical mucus and narrow aperture and passageway of the endocervix observed in cetaceans appear to largely occlude the opening of the uterus [11; 23]. Future studies that investigate the relationship between vaginal length, vaginal fold complexity, and cervical conformation are warranted.

Vaginal modifications could also be shaped by post-copulatory sexual selection and the coevolution of male and female reproductive anatomy [5; 67–68]. Vaginal folds might be concentrated in the cranial half of the vagina, because ejaculation occurs proximate to the cervix and deep to the vaginal orifice (T. Robeck, pers. comm.). Since the distal tips of vaginal folds projected caudally within the lumen of a vagina and generally decreased in size from cranial to caudal direction, like a funnel, they may restrict sperm from entering the cranial vagina or upper reproductive tract rather than retention. Future studies that use *in vivo* vaginal endoscopy of recently mated cetaceans or physical models might be able to illuminate whether vaginal folds form physical barriers to seawater and sperm movement; this can be accomplished by examining and distinguishing where seawater and sperm pool within the vagina or vaginal model.

Assessment of the relationship between vaginal complexity and testes size or penile morphology (testes size and penile length are correlated in cetaceans; [69–70]) could provide evidence of a role of vaginal folds in sexual selection. For example, oviduct length was positively correlated with testes weight and with sperm characteristics across 33 genera of mammals [71]. Penile morphological diversity has not been well documented among cetaceans. Future studies of how penile morphology relates to vaginal fold shape, length, or positioning, and how deep the penis penetrates the vagina and possibly the cervix during copulation are needed to further explore the sexual selection functional hypothesis of vaginal folds.

Only three species- pygmy sperm whales (*Kogia breviceps*), dwarf sperm whales (*Kogia sima*), and harbor porpoises (*Phocoena phocoena*)- were consistently located above the body size regression line for both vaginal length and cumulative vaginal fold length (Fig 3). These three species all invest heavily in sperm competition, as supported by their large relative testes sizes [72]. Life history and socio-biological factors could explain why these three species were consistently located above the regression lines, unlike other species with large relative testes sizes. For example, pygmy sperm whales, dwarf sperm whales, and harbor porpoises have been identified in the “fast end” of the life history continuum for cetaceans [73–74]. Reproductive tract characteristics that favor the viability of sperm in potentially lethal aquatic environments (e.g., physical barriers to water flow) are particularly beneficial for species with frequent reproduction.

The data demonstrate that vaginal length was not a significant predictor of cumulative vaginal fold length. This may be because the two characters function differently to restrict seawater and/or semen to the caudal vaginal region (i.e., physical distance and physical barrier). Cumulative vaginal fold length was selected as a variable because it was a straightforward and quantitative measure of the extent of obstruction of the vaginal lumen. However, other features of vaginal folds, such as thickness, shape, or number—as well as other features of the entire reproductive tract, such as the lengths of various chambers—could be informative in determining function. Although data were not obtained for all 90 extant cetacean species, the data are representative of 11 genera across the cetacean phylogeny. The data provide a robust indication of taxon-wide patterns that can be further expanded to advance our understanding of the evolution of genital morphology.

## Conclusions

Although vaginal folds have been reported in several cetacean species [14], quantitative measurements of vaginal morphology have not been collected for most species, nor measured using consistent landmarks. This lack of information has hindered the ability to systematically assess the function(s) of vaginal folds in cetaceans. Although vaginal length scaled isometrically with body length, cumulative vaginal fold length scaled with significant positive allometry. Since most variance in the regressions was not explained by body size and the factors were not predictors of each other, vaginal fold diversity may be maintained by additional natural and/or sexual selection pressures. This study lays the foundation for tests of functionality that will determine if vaginal folds are an example of specialized adaptations to aquatic living and/or relate to sexual selection.

## Supporting information

S1 Fig Regression of cumulative vaginal fold length on number of vaginal folds. The non-phylogenetically controlled residuals of cumulative vaginal fold length on total body length were used. The solid black line indicates the line of best-fit from a phylogenetic reduced major axis regression (R^2^ = 0.113, *t* = 10.997, *df* = 19, *P* < 0.01).
(TIF)

S1 Table Counts and measurements of the specimens. Measurements were not scaled by body length. The U.S. state (or country for New Zealand) where each specimen stranded is listed. The median is listed in parentheses when it varies from the mean.
(DOCX)
