# Supplementary figures and images for: Patterns of cetacean vaginal folds yield insights into functionality
Source: PLoS One. 2017 Mar 31;12(3):e0175037. doi: 10.1371/journal.pone.0175037 (PMC5376333; doi:10.1371/journal.pone.0175037)

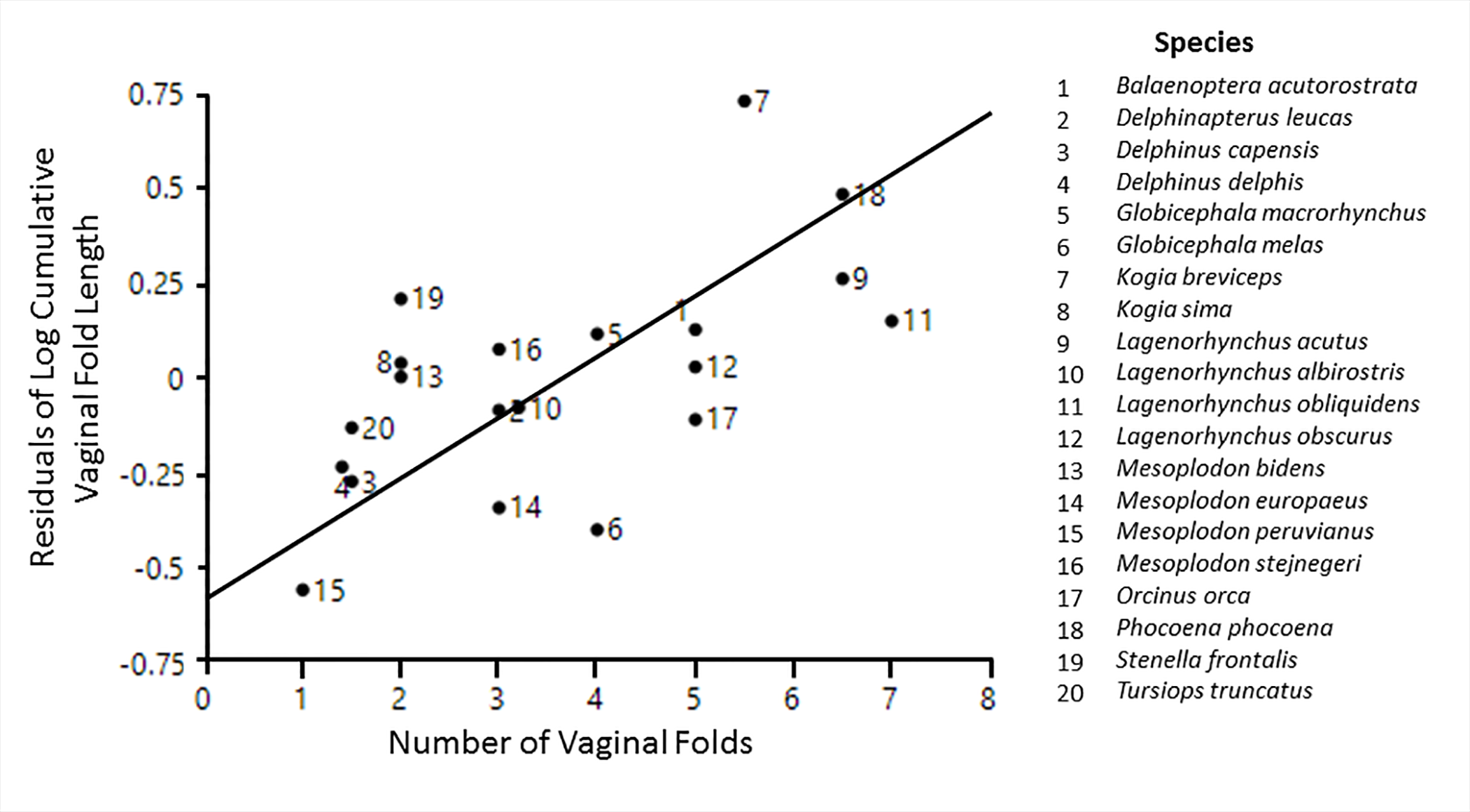

Supplement: S1 Fig — The non-phylogenetically controlled residuals of cumulative vaginal fold length on total body length were used. The solid black line indicates the line of best-fit from a phylogenetic reduced major axis regression (R2 = 0.113, t = 10.997, df = 19, P < 0.01). (TIF) [file pone.0175037.s001.tif]
