# Supplementary material for: Patterns of cetacean vaginal folds yield insights into functionality
Source: PLoS One. 2017 Mar 31;12(3):e0175037. doi: 10.1371/journal.pone.0175037 (PMC5376333; doi:10.1371/journal.pone.0175037)
Supplement: S1 Table — Measurements were not scaled by body length. The U.S. state (or country for New Zealand) where each specimen stranded is listed. The median is listed in parentheses when it varies from the mean. (DOCX) [file pone.0175037.s002.docx]

**S1 Table. Counts and measurements of the specimens.** Measurements were not scaled by body length. The U.S. state (or country for New Zealand) where each specimen stranded is listed. The median is listed in parentheses when it varies from the mean.

| **Species** | **No. of Sexually Mature Specimens** | **No. of Sexually Immature Specimens** | **Mean No. Vaginal Folds** | **Mean (±SD) Vaginal Length (mm)** | **Mean (±SD) Cumulative Vaginal Fold Length (mm)** | **Stranding Location(s)** | |
| --- | --- | --- | --- | --- | --- | --- | --- |
| **Family Balaenoptera** |  |  |  |  |  |  |  |
| *Balaenoptera acutorostrata* | 0 | 1 | 5. 0 **±** 0 | 241.4 **±** 0 | 71.0 **±** 0 | Virginia |  |
| **Family Delphinidae** |  |  |  |  |  |  |  |
| *Delphinus capensis* | 2 | 0 | 1.5 **±** 0.7 | 117.0 **±** 37.9 | 17.5 ± 0.7 | California |  |
| *Delphinus delphis* | 3 | 2 | 1.4 **±** 0.5  (1.0) | 128.2 ± 18.6  (123.5) | 17.8 ± 6.5  (16.0) | Massachusetts, North Carolina |  |
| *Globicephala macrorhynchus* | 1 | 0 | 4.0 **±** 0 | 209.0 **±** 0 | 64.0 **±** 0 | Florida |  |
| *Globicephala melas* | 0 | 1 | 4.0 **±** 0 | 180.8 **±** 0 | 12.2 **±** 0 | Massachusetts |  |
| *Lagenorhynchus acutus* | 1 | 0 | 5.0 **±** 0 | 184.5 **±** 0 | 37.0 **±** 0 | Massachusetts |  |
| *Lagenorhynchus albirostris* | 1 | 0 | 7.0 **±** 0 | 209.0 **±** 0 | 51.0 **±** 0 | Massachusetts |  |
| *Lagenorhynchus obliquidens* | 1 | 3 | 6.5 **±** 1.3 | 157.8 **±** 47.3  (153.5) | 55.0 **±** 14.0  (54.0) | California, Oregon |  |
| *Lagenorhynchus obscurus* | 3 | 0 | 3.3 ± 1.2 | 77.6 ± 3.9 | 24.0 ± 2.6 | New Zealand |  |
|  |  |  | (4.0) | (78.7) | (23.0) |  |  |
| *Orcinus orca* | 0 | 2 | 5.0 **±** 5.7 | 278.8 ± 150.5 | 34.0 ± 21.2 | Alaska, New Zealand |  |
| *Stenella frontalis* | 0 | 1 | 2.0 **±** 0 | 99.0 ± 0 | 48.0 ± 0 | North Carolina |  |
| *Tursiops truncatus* | 9 | 3 | 1.5 ± 0.8 (1.0) | 138.1 ± 42.7  (131.5) | 25.4 ± 9.2  (25.5) | Florida, Texas, Virginia |  |

| **Family Kogiidae** |  |  |  |  |  | |  | |  |
| --- | --- | --- | --- | --- | --- | --- | --- | --- | --- |
| *Kogia breviceps* | 2 | 0 | 5.5 ± 2.1 | 765.0 ± 75.0 | | 219.5 ± 119.5 | | Florida, North Carolina | |
| *Kogia sima* | 0 | 1 | 2.0 **±** 0 | 375.0 ± 0 | | 32.0 ± 0 | | Florida | |
| **Family Monodontidae**  *Delphinapterus leucas*  **Family Phocoenidae** | 1 | 0 | 3.0 **±** 0 | 355.0 ± 0 | | 39.0 ± 0 | | Alaska | |
| *Phocoena phocoena* | 12 | 4 | 6.5 ± 2.7  (7.0) | 194.2 ± 61.3  (209.2) | | 80.2 ± 35.7  (85.9) | | Alaska, California, Massachusetts, Oregon, Washington | |
| **Family Ziphiidae** |  |  |  |  | |  | |  | |
| *Mesoplodon bidens* | 1 | 0 | 2.0 **±** 0 | 662.0 ± 0 | | 53.0 ± 0 | | Massachusetts | |
| *Mesoplodon europeaus* | 1 | 1 | 3.0 **±** 0 | 236.6 ± 12.9 | | 21.0 ± 7.1 | | Florida, North Carolina | |
| *Mesoplodon peruvianus* | 0 | 1 | 1.0 **±** 0 | 243.8 ± 0 | | 10.0 ± 0 | | California | |
| *Mesoplodon stejnegeri* | 1 | 0 | 3.0 **±** 0 | 617.0 ± 0 | | 70.0 ± 0 | | Oregon | |
